# IL-6 predicts organ dysfunction and mortality in patients with multiple injuries

**DOI:** 10.1186/1757-7241-17-49

**Published:** 2009-09-27

**Authors:** Michael Frink, Martijn van Griensven, Philipp Kobbe, Thomas Brin, Christian Zeckey, Bernhard Vaske, Christian Krettek, Frank Hildebrand

**Affiliations:** 1 Trauma Department, Hannover Medical School, Carl-Neuberg-Str 1, 30625 Hannover, Germany; 2 Ludwig Boltzmann Institute for Experimental and Clinical Traumatology, Donaueschingenstraße 13, A-1200 Vienna, Austria; 3 Department of Trauma Surgery, University Hospital Essen, Hufelandstr 55, 45122 Essen, Germany; 4 Center for Biometry, Hannover Medical School, Carl-Neuberg-Str 1, 30625 Hannover, Germany

## Abstract

**Background:**

Although therapeutic concepts of patients with major trauma have improved during recent years, organ dysfunction still remains a frequent complication during clinical course in intensive care units. It has previously been shown that cytokines are upregulated under stress conditions such as trauma or sepsis. However, it is still debatable if cytokines are adequate parameters to describe the current state of trauma patients. To elucidate the relevance of cytokines, we investigated if cytokines predict development of multiple organ dysfunction syndrome (MODS) or outcome.

**Methods:**

A total of 143 patients with an injury severity score ≥ 16, between 16 and 65 years, admitted to the Hannover Medical School Level 1 Trauma Center between January 1997 and December 2001 were prospectively included in this study. Marshall Score for MODS was calculated for at least 14 days and plasma levels of TNF-α, IL-1β, IL-6, IL-8 and IL-10 were measured. To determine the association between cytokine levels and development of MODS the Spearman rank correlation coefficient was calculated and logistic regression and analysis were performed.

**Results and Discussion:**

Patients with MODS had increased plasma levels of IL-6, IL-8 and IL-10. IL-6 predicted development of MODS with an overall accuracy of 84.7% (specificity: 98.3%, sensitivity: 16.7%). The threshold value for development of MODS was 761.7 pg/ml and 2176.0 pg/ml for mortality during the in patient time.

**Conclusion:**

We conclude that plasma IL-6 levels predict mortality and that they are a useful tool to identify patients who are at risk for development of MODS.

## Background

During the last decades, improvement of therapeutic concepts has decreased trauma related fatalities [1]. Organ dysfunction is still a frequent and severe complication during clinical course and the most common cause for late fatalities following major trauma. Although the survival rate of patients with multiple injuries improved during the last decades the frequency of development of organ dysfunction has not changed [2,3]. The mortality of patients developing multiple organ dysfunction syndrome (MODS) following severe injuries is still 50% [4-6]. For adequate treatment, it would be desirable to identify patients with a high risk for posttraumatic complications in the early clinical course. The evaluation of clinical state and prognosis still remains one of the greatest challenges during treatment of patients suffering from multiple injuries. Many clinical parameters such as blood pressure, pH or heart rate failed to assess the posttraumatic situation [7].

Several clinical studies have demonstrated that increased cytokine plasma levels are correlated with MODS, severity of injury, as well as mortality [8-13]. While plasma IL-6 levels were able to predict outcome in a murine sepsis model [14], the data in humans is still controversial [12,15-17]. Thus, it was the purpose of our study to correlate plasma cytokine levels with MODS and mortality and determine threshold values of these cytokines for development of MODS. We hypothesized that plasma cytokines levels can predict MODS and mortality in humans following major trauma. To test this hypothesis we correlated plasma levels of IL-1β, IL-6, IL-8, IL-10 and TNF-α as well as traditional parameters such as lactate, platelets and base excess with MODS and mortality.

## Methods

### Inclusion and exclusion criteria

Polytraumatized patients between the ages of 16 and 65 years who were admitted to Hannover Medical School Level 1 Trauma Center between January 1997 and December 2001 were prospectively included in this study. Patients with an injury severity score <16 points were excluded. In addition, patients with a history of steroid use, anti-inflammatory treatment or hormone replacement therapy were excluded. Patients with malignancies or chronic diseases of the liver, kidneys or lung were also excluded (Table 1).

**Table 1:** Demographic data of included patients; *p < 0.05 MODS vs. No MODS.

**Parameter**	**MODS**	**No MODS**	**All patients**
Patients [n]	24	119	143
Age [years]	40.0 ± 3.6	36.3 ± 1.4	36.9 ± 1.3
Sex [m:f]	7:1*	2.5:1	2.9:1
GCS	9.0 ± 1.0	10.5 ± 0.5	10.2 ± 0.4
ISS	28.5 ± 2.1	24.5 ± 0.7	25.1 ± 0.7
Mortality [%]	54.2	6.7	14.7

### Ethical approval and informed consent

The study was approved by the Ethical Committee of the Hannover Medical School, Hannover, Germany. Informed consent was obtained from all patients (or their relatives) included in this study.

### Pattern and severity of injury

Additionally, the abbreviated injury scale was determined after a trauma scan (CT scan of head, cervical spine, thorax, abdomen and pelvis) and severity of injury was calculated using the injury severity score (ISS).

### Clinical parameter and outcome evaluation

Patients were carefully examined at 7 AM and blood (10 ml) was daily collected for routine analysis and cytokine measurement (TNF-α, IL-1β, IL-6, IL-8, IL-10). Plasma cytokines were determined using a commercially available kit (Immulite^® ^System: Random Access Immunoassay Analyser; DPC-Biermann, Bad Nauheim, Germany) following the manufacturer's instructions. The results of clinical examination and blood chemistry (C-reactive protein [CRP], platelets, lactate and base excess) were recorded up to 14 days after admission (Table 2).

**Table 2:** Correlation coefficient of laboratory parameters and development of MODS.

**Parameter**	**Correlation coefficient**
IL-1β	0.00
IL-6	0.35*
IL-8	0.53*
IL-10	0.31*
TNF-α	0.32*
CRP	0.27*
Platelets	-0.32*
Lactate	0.37*
Base Excess	0.11*

* p < 0.01

Diagnosis of sepsis was made according to the criteria of the Consensus Conference of the *American College of Chest Physicians *(ACCP) and the *Society of Critical Care Medicine *(SCCM) [18] on at least two consecutive days [19]. MODS was diagnosed using the score of Marshall et al [20]. This score has been shown to be the most reliable score for diagnosis of MODS [21]. As previously described, a manifest MODS was considered when the score was >12 points on two consecutive days or at least three days during the observed period [22].

### Patient management and treatment

After admission, all patients received an arterial and a central venous line. A standardized clinical examination, a focused assessment with sonography for trauma (FAST) and at least chest and pelvic x-rays were performed. After diagnostics in the emergency room, a trauma scan (CT-scan of head, cervical spine, chest, abdomen and pelvis) was accomplished. Results were analyzed by an attending radiologist and an attending trauma surgeon. At time of admission to the intensive care unit (ICU), the clinical examination and FAST were repeated. Included patients were treated by physicians who are not involved in this study.

### Subgroup analysis

Depending on fulfillment of the MODS criteria, patients were divided into two groups, those with and without multiple organ dysfunction syndrome (MODS, no MODS).

### Statistics

Statistical analysis was performed using SPSS, version 15 (SPSS, Chicago, IL, USA). Results from descriptive analyses are expressed as mean ± standard error of the mean. Statistical significance was assumed where probability values p of less than 0.05 were obtained. Comparison between groups was performed using one-way analysis of variances (ANOVA) followed by the Tukey test. The Spearman rank correlation coefficient was used to determine the connection between cytokine levels and development of MODS. Additionally we performed an analysis of the relationship between the plasma cytokine concentrations and complications using logistic regression for identifying relevant parameters as well as a receiver operating characteristic (ROC) curve analysis for validation of Il-6 as a predictive marker.

## Results

### Demographics

A total of 143 patients (106 males and 37 females) were included in this study. Age and ISS were comparable in all analyzed subgroups. Additional characteristics are shown in Table 1.

### Pattern and severity of injury

Pattern and severity of injury were comparable in all analyzed groups. Furthermore, injury severity and pattern described by AIS (abbreviated injury scale; data not shown) and ISS (Table 1) showed no significant difference in all analyzed subgroups.

### MODS influence on plasma cytokines

#### TNF-α

While plasma levels of TNF-α were comparable on day 1 in patients with and without MODS, a steady increase was observed in the MODS group (p < 0.05).

#### IL-1β

During the observed period, MODS did not influence plasma levels of IL-1β (p > 0.05).

#### IL-6

During the entire observation period, patients with MODS had higher plasma levels of IL-6 than patients without MODS. Differences were more distinct during the first week (p < 0.05).

#### IL-8

Plasma levels of IL-8 were significantly higher in MODS patients than in patients who did not have MODS with a distinct difference at day 1 (p < 0.05).

#### IL-10

MODS patients had increased plasma levels of IL-10 on days 1 to 3 and days 9 to 14 compared with patients without MODS (p < 0.05).

### MODS influence on other plasma parameters

#### CRP

MODS patients had increased plasma CRP levels on days 4 to 14 (p < 0.05).

#### Platelets

During the entire observation period, patients with MODS had higher platelet counts than patients without MODS (p < 0.05).

#### Lactate

Patients with organ dysfunction had higher plasma lactate levels as compared to patients with uneventful recovery on days 1 to 14 (p < 0.05).

#### Base excess

Base excess was elevated in patients with MODS on days 2, 3 and 13 (p < 0.05).

### Correlation between cytokines and MODS

Besides IL-1β all analyzed cytokines showed a significant correlation between cytokine plasma concentration and development of MODS. The correlation coefficients for all analyzed cytokines are shown in Table 2.

### Correlation between laboratory values and MODS

All tested parameters correlated with development of MODS (see Table 3).

**Table 3:** Specifity, sensitivity and accuracy of laboratory markers.

**Parameter**	**Specificity**	**Sensitivity**	**Overall accuracy**
IL-6	98.3%	16.7%	84.7%*
IL-8	n.d.	n.d.	n.d.
IL-10	n.d.	n.d.	n.d.
TNF-α	n.d.	n.d.	n.d.
CRP	n.d.	n.d.	n.d.
Platelets	98.3%	8.3%	83.2%*
Lactate	99.1%	8.7%	83.9%*
Base Excess	n.d.	n.d.	n.d.
IL-6+Lactate	97%	17%	84.7%*
IL-6+Platelets	98%	21%	84.6%*
IL-6+Lactate+Platelets	97%	26%	85.4%*

### Sensitivity, specificity and overall accuracy

For all cytokines with a correlation coefficient >0.26, sensitivity, specificity and overall accuracy were calculated. The sensitivity for IL-8, IL-10, TNF-α, CRP and base excess was 0%. The specificity of IL-6 was 98.3% while sensitivity was only 16.7% (overall accuracy 84.7%). Combination of IL-6 with platelets and lactate improved sensitivity and specificity (Table 3).

### Critical value of IL-6 for MODS development

Since IL-6 was the best parameter for predicting the development of posttraumatic MODS we calculated a threshold value at which the probability of MODS development is >50%. At a plasma IL-6 concentration of 761.7 pg/μl, >50% patients developed MODS. Injury pattern and severity is shown in Table 4.

**Table 4:** Injury pattern and severity of patients with systemic IL-6 levels above the critical value for the development of MODS.

**AIS**_head/neck_	**AIS**_face_	**AIS**_chest_	**AIS**_abdomen_	**AIS**_Extremity_	**AIS**_soft tissue_	**ISS**
2.8 ± 1.1	0 ± 0	3.5 ± 0.8	3.0 ± 1.3	3.0 ± 0.4	1.0 ± 0.0	28.2 ± 11.9

### Prognostic value of IL-6 for mortality in the early clinical course

At day 1 plasma IL-6 levels had a specificity of 100% while the sensitivity was 28.6%. The overall accuracy was 86.1%. At day 2, similar values were detected (specificity: 97.8%, sensitivity: 19.0%, overall accuracy: 83.2%) The IL-6 threshold value for mortality during the in-patient time was 2176.0 pg/ml.

### ROC curve analysis

**Figure 1:**
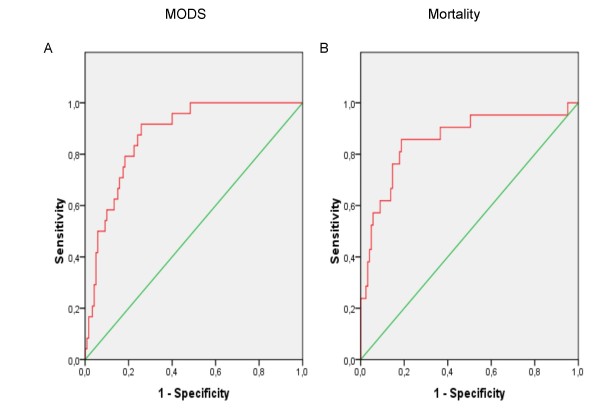
**ROC curve analysis of IL-6 for the prediction of MODS (A) and mortality (B)**.

The ROC curve analysis for IL-6 for predicting MODS and mortality are shown in Fig. 1. The areas under the curve for MODS and mortality are, respectively, 0.874 (SE 0.03; 95% confidence interval [CI] 0.8110.937), 0.858 (SE 0.05; 95% CI 0.759-0.956).

## Discussion

As previously published, we have investigated the plasma levels of common parameters (CRP, platelets, lactate and base excess) and various cytokines such as TNF-α, IL-1β, IL-6, IL-8, and IL-10 in patients suffering from major trauma[13] Besides IL-1β, all cytokines showed higher levels in patients matching the MODS criteria and correlated with MODS. Patients with MODS had no greater severity of injury or a different injury pattern [13]. IL-6 not only showed the best correlation but predicted development of MODS with an overall accuracy of 84.7%. In addition, IL-6 was the best parameter in predicting mortality (overall accuracy 86.1% at day 1 and 83.2% at day 2).

The fact that patients with MODS had higher cytokine plasma levels is consistent with our previously published results in the same population as well as observations from other investigators [8,12,13,15,23]. However, to the best of our knowledge, this is the first study providing a threshold value of IL-6 for development of organ dysfunction and mortality.

The authors are aware of the limitations of the present study due to an inhomogeneous population. There are several factors influencing systemic cytokine levels (i.e. number of blood transfusions, gender, genetic polymorphisms) that cannot be controlled due to the design of the study [13,24,25].

Since trauma has a huge socioeconomic impact [26] and sepsis and MODS increase costs of treatment in trauma patients [27] it is necessary to identify patients susceptible for development of MODS in early clinical course to adjust therapeutic interventions. The concept of damage control surgery is based on studies investigating further damage by operations in the early clinical phase [10]. Though the outcome of patients has improved during recent years, defined parameters predicting posttraumatic complications are still lacking.

The present study shows increased plasma IL-6 levels in patients with MODS as compared to patients with uneventful recovery. These findings confirm studies from other investigators [12,15]. However, in several studies a correlation between IL-6 and MODS was not proven [16,17]. Since in both studies only 16 and 13 patients, respectively, were included lack of a correlation between plasma IL-6 levels and MODS could be due to the small number of patients. In the present study, IL-6 was the best parameter for predicting development of MODS as compared to other cytokines. Since IL-6 had the highest specificity and overall accuracy we calculated a threshold value above which the development of MODS is likely. This critical value may be of high relevance in further treatment of polytraumatized patients. In the present study, we showed that IL-6 predicts outcome in patients following major trauma. Thus, we confirmed studies from a murine septic shock model showing that IL-6 predicts outcome in the early phase of sepsis [14,28]. In humans, a correlation between high plasma IL-6 levels and outcome was shown in pediatric patients with major head injury [29]. Martin et al. showed that elevated plasma levels were associated with fatal outcome in septic shock stage [30]. Additionally, increased IL-6 values were an indicator of the development of a nosocomial infection in trauma patients. However, to our knowledge this is the first study describing a critical value of IL-6 for development of MODS and predicting mortality.

Patients matching the MODS criteria had higher plasma IL-8 levels than patients without complications. We could determine a correlation between systemic IL-8 concentrations and the MODS score. This finding is in accordance with data from other investigators who also show an association between organ dysfunction and increased plasma IL-8 levels in patients with major injury [11]. Furthermore, patients with established diagnosis of adult respiratory distress syndrome, a common posttraumatic complication [31], showed elevated IL-8 levels in bronchoalveolar lavage fluid [8]. In the performed logistic regression analysis, we could show a correlation between MODS and IL-8 levels, but IL-8 was not able to identify patients suffering from MODS. Thus, IL-8 seems not to be an adequate parameter regarding the development of MODS. Since IL-8 showed an association to thoracic trauma [8,9] its role in this particular injury needs to be evaluated in further studies.

In the present study patients with MODS had higher IL-10 plasma levels in the early clinical course. Elevated systemic IL-10 levels correlated with MODS but were not of value predicting the development of organ failure. Neidhard et al. showed that increased plasma IL-10 levels are not only associated with posttraumatic complications but also with severity of injury [23]. Since IL-10 is an anti-inflammatory cytokine, elevated levels can be estimated as a compensatory anti-inflammatory response to prevent possible harmful hyperinflammation.

Although IL-10 levels were increased in patients with MODS, studies have shown that elevated levels may contribute to augmented organ dysfunction in trauma patients [32]. However, IL-10 failed to predict development of organ dysfunction in the present study.

Patients with MODS did not have higher levels of IL-1β as compared to patients without MODS. This is in accordance with findings from other studies in which IL-1-β was not increased during response to septic shock secondary to generalized peritonitis [33]. Contrary to these results, other investigators proved an association between IL-1β and with an increased mortality rate and an increased risk for subsequent ARDS and MOF in patients following major vascular surgery, trauma or hemorrhagic shock [12]. These conflicting results may be due to the short half-life (6 min) of this mediator [34]. However, our results indicate that IL-1β is not correlated with MODS and is therefore not a useful parameter for predicting posttraumatic organ dysfunction.

In the present study, patients suffering from organ dysfunction had increased plasma TNF-α levels as compared to patients without MODS. Similar results were shown by other investigators for trauma patients [35], as well as in burn patients [36]. Although there is an association between TNF-α and MODS, this pro-inflammatory cytokine failed to predict development of MODS investigated in the logistic regression analysis.

As previously described, the traditional parameters for describing the status of patients following trauma or with major surgery were associated with organ dysfunction [7,37,38]. Although all investigated parameters failed to predict mortality or development of MODS, in combination with IL-6, they improved the sensitivity, specificity and overall accuracy as compared to IL-6 alone. Although secretion of CRP is induced by IL-6[39] in the present study, CRP failed to correlate with the development of MODS.

## Conclusion

In the current study we demonstrated the correlation between various cytokines and MODS in polytraumatized patients. We determined a threshold value for IL-6 for predicting development of MODS and predicting mortality. This will help to identify patients in the early clinical period who are susceptible to develop organ dysfunction. This is important insofar as these patients require special therapeutic concepts such as damage control surgery.

## List of abbrevations

ARDS: adult respiratory distress syndrome; CRP: C-reactive protein; IL: interleukin; ISS: injury severity score; ml: millilitre; MOF: multi organ failure; MODS: multi organ dysfunction syndrome; pg: picograme; TNF: tumor necrosis factor

## Conflict of interests

The authors declare that they have no competing interests.
